# Implementation of laparoscopic surgery for endometrial cancer: work in progress

**Published:** 2016-03-28

**Authors:** AAS Van den Bosch, HJMM Mertens

**Affiliations:** Junior-resident, Zuyderland Medical Center Sittard.; Gynecologist, Zuyderland Medical Center Sittard.

**Keywords:** Endometrial cancer, laparoscopy, open surgery, minimal invasive treatment, implementation, technique

## Abstract

**Aim of the study:**

Even in oncology, minimal invasive surgery is introduced. We describe the introduction of this new surgical technique in a teaching hospital. The objective was to compare factors that have influenced the choice between laparoscopic versus open surgery in patients with endometrial cancer.

**Methods:**

We retrospectively analysed all patients with endometrial cancer between 2010 and 2014. The different factors we compared were age, weight, histopathology, uterine size, serum CA-125 level and FIGO stage.

**Results:**

The choice for laparoscopic surgery gradually increased in the years after introduction. An analysis from data’s from 2010-2014 showed that all discriminative factors did not significantly influence the choice between laparoscopic versus open surgery, besides endometrial histopathology (tumour grade and tumour histology).

**Conclusion:**

Both laparoscopy and open surgery are safe approaches in treating endometrial cancer. The surgical procedure itself did not have impact on survival. In all patients with endometrial cancer, even in obese patients, we recommend laparoscopy to reduce postoperative complications.

## Introduction

Endometrial carcinoma is the most common malignancy of the female genital tract (IKNL, 2011). The first laparoscopic treatment for endometrial cancer was described by Childers et al. in 1993 who reported two cases of laparoscopic- assisted vaginal hysterectomy (LAVH) for the treatment of endometrial cancer. Since then, indications for laparoscopic surgery are rising. It took almost two decades to introduce laparoscopy as a routine treatment in gynaecologic cancer. In 2010 a multicentre prospective trial in the Netherlands about the possibility of laparoscopy in gynaecologic oncology was published in Lancet Oncology (Mourits et al., 2010).

Implementation of laparoscopic surgery for endometrial cancer is described by Hauspy et al. (2010). Discriminating factors in the choice for laparoscopy versus laparotomy for any indication are age, weight, co-morbidity, histopathology, uterine size, serum level of CA-125 and FIGO stage. Mourits and co-authors observed no difference in major complication rate between both treatment options. Although they showed a difference in hospital stay, less pain and quicker resumption of daily activities if the operation was performed by a skilled surgeon (Bijen et al., 2010; Mourits et al., 2010).

Before 2009, all patients with endometrial cancer in the Orbis Medical Centre in Sittard (OMC). were treated by a total hysterectomy with bilateral salpingo-oophorectomy after an abdominal medial incision. Only since 2010 laparoscopy is performed in women with endometrial carcinoma

In a first period only women with grade 1 or 2 endometrioid endometrial carcinoma were treated by laparoscopy. Later on the indication was extended, due to positive experiences and increasing confidence in the technique. The introduction of a relatively new procedure for new indications was not simple and always straightforward, mainly because the gynaecological oncologist was only used to abdominal laparotomy.

The aim of this study was to analyse the outcomes after introduction of minimal invasive surgery treatment in patients having endometrial cancer in a teaching hospital in The Netherlands. The objective was to compare factors that have influenced the choice between laparoscopic versus open surgery in patients with endometrial cancer.

## Methods

All women diagnosed with endometrial carcinoma in the Orbis Medical Centre in Sittard (OMC) between January 2010 until December 2013 were divided into three groups depending on their surgical treatment. The OMC is a teaching, non-university hospital in the South of The Netherlands. Uterine and ovarian cancer patients can be treated in this hospital; patients with vulvar or cervical cancer are referred to specialized centres.

There were three groups of patients: patients that were planned and underwent a laparoscopic procedure (group I); patients planned for a laparoscopy but in whom the procedure was converted into a laparotomy (group II) and patients that were planned for a laparotomy (group III).

Data from the electronic medical patient files were analysed with IBM SPSS Statistics 19 SP2. Continuous variables were analysed using ANOVA. Post hoc test Tukey HSD was used to compare different groups. Categorical variables were analysed using a chi-squared test. Odds ratios were determined by logistic regression to see which factors (age ≥ 65 years, BMI, cardiac history, pulmonal history, diabetes mellitus, coagulation disorder, previous abdominal surgery, number of previous abdominal surgery) infuenced the choice for laparoscopy or laparotomy. A p-value < 0.05 was considered as signifcantly different. 

## Results

Overall, 110 women were diagnosed having endometrial cancer. Fourteen patients (12.7%) did not undergo any surgery because of metastatic disease (n = 6), cardiac or pulmonary co-morbidity (n = 2) and 6 patients refused surgery because of advanced age (n = 6). In 2 patients, endometrial cancer was unexpectedly diagnosed postoperatively. They underwent a vaginal hysterectomy because of vaginal prolapse (n = 1) or urinary incontinence (n = 1).

In total, 94 women were planned for surgery because of endometrial cancer. Twenty-fve patients where operated in 2010 (26.6%), 26 patients in 2011 (27.7%), 21 patients in 2012 (22.3%) and 22 patients in 2013 (23.4%).

Of these patients, 18 (19.2%) underwent laparoscopic surgery (group I). In 5 patients (5.3%) the laparoscopy was converted into a laparotomy (group II). Seventy-one patients (75.5%) were primarily planned for a laparotomy (group III). 

Of all laparoscopic procedures, 21.7% were converted (group II). The reasons for conversion where an immobile uterus with many adhesions (n = 2), too little space for laparoscopic coagulation (n = 2) or a large uterus myomatosus (n = 1). The conversion rate was not signifcantly different over the years (P = 0.250).

In high-risk endometrial cancer, a laparotomy for staging or debulking is always performed. For that reason, patients that were primarily planned for laparotomy (group III) were further subdivided into two subgroups based on accepted indications for laparoscopic treatment for endometrial cancer. Patients that were planned for a laparotomic procedure with grade I and grade II endometrioid adenocarcinomas are known as group IIIa versus patients with non-endometrioid and poorly differentiated endometrioid tumours, known as group IIIb.

**Fig. 1:**
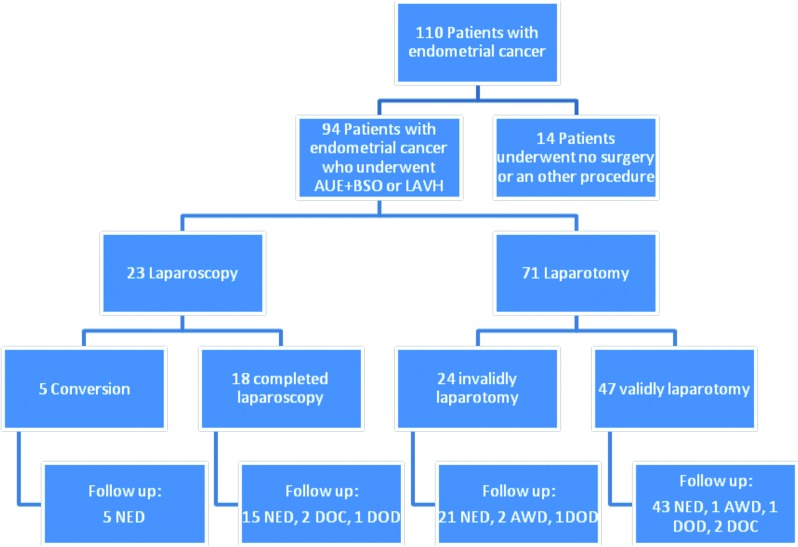
— Patient selection This figure shows all patients that were operated for endometrial cancer in this study. In the bottom line, the follow up of these patients is added (AUE = abdominal uterus extirpation, BSO = bilateral salpingo-oophorectomy, LAVH = laparoscopic assisted vaginal hysterectomy, NED = no evidence of disease; AWD = alive with disease; DOC = death other cause, DOD = death on disease.

Of all patients (45%) that could have been planned for a laparoscopic procedure, 18 patients (43%) were planned for laparoscopy, 24 patients (57%) still got a laparotomy (group IIIa) (Fig. 1).

### Pre-operative patients’ characteristics (Table I)

**Table I:** — Pre-operative baseline characteristics in patients that were operated because of endometrial cancer in this study (laparoscopy = group I; conversion = group II; laparotomy invalid = group IIIa; laparotomy valid = group IIIB).

		Laparoscopy (n = 18)	Conversion (n = 5)	Laparotomy (n = 71)		p-value
				Laparotomy invalid (n = 24)	Laparotomy valid (n = 47)	
Mean age during diagnosis, years (95%-CI)		64.3 (54.3-74.2)	61.5 (55.2-67.9)	64.7 (60.3-69.2)	66.3 (63.4-69.4)	0.41
Mean BMI, kg/m2 (95%-CI)		31.9 (27.6-36.2)	37.6 (29.3-45.8)	28.4 (26.4-30.3)	28.4 (26.5-30.3)	0.12
Postmenopausal		14 (77.8%)	5 (100%)	21 (87.5%)	42 (89.4%)	0.91
Cardiac history		9 (50.0%)	1 (20.0%)	9 (37.5%)	23 (48.9%)	0.36
Pulmonal history		2 (11.1%)	0	3 (12.5%)	1 (2.1%)	0.34
Diabetes Mellitus		0	2 (40.0%)	3 (12.5%)	11 (23.4%)	0.30
Coagulation disorder		0	0	2 (8.3%)	2 (4.3%)	0.68
Previous abdominal surgery		7 (38.9%)	1 (20.0%)	8 (33.3%)	21 (44.7%)	0.40
	Mean number of previous abdominal surgeries (95%-CI)	0.7 (0.0-1.4)		1.7 (0.6-2.8)	1.4 (1.0-1.8)	0.14
Preoperative type of tumour						< 0.01
	Endometrioid Adenocarcinoma	16 (88.9%)	4 (80.0%)	24 (100%)	34 (72.3%)	
	Serous Carcinoma	0	0	0	11 (23.1%)	
	Unknown	2 (11.1%)	1 (20.0%)	0	2 (4.3%)	
Preoperative grade of tumour						< 0.01
	Good	10 (55.6%)	3 (60.0%)	24 (100%)	0	
	Moderate	6 (33.3%)	1 (20.0%)	0	26 (55.3%)	
	Poor	0	0	0	17 (36.2%)	
	Unknown	2 (11.1%)	1 (20.0%)		4 (8.5%)	
Preoperative CA-125						0.58
	< 21U/ml	11 (61.1%)	5 (100%)	20 (83.3%)	33 (70.2%)	
	> 21U/ml	4 (22.2%)	0	4 (16.7.0%)	9 (19.1%)	
	Unknown	3 (17.7%)	0		5 (10.6%)	

#### Age

The mean age of patients having endometrial cancer was 65.4 years (95%-CI 54.9-75.9). In group I, II, IIIa and IIIB the mean ages were respectively 64.3 years (95%-CI 54.3-74.2), 61.5 years (95%-CI 55.2-67.9), 64.7 years (95%-CI 60.3-69.2) and 66.3 years (95%-CI 63.4-69.4). The differences between these groups were not significant.

#### BMI

The mean BMI of patients having endometrial cancer was 30.2 kg/m2 (95%-CI 22.6-37.8). The BMI was not significantly different between the three groups.

The majority of patients had a BMI above 27 (74 patients or 79%). A BMI of more than 27 was found in 15 patients (94%) of group I, in 5 patients (100%) of group II, in 21 patients (86%) of group IIIa and in 28 patients (65%) of group IIIb. The BMI did not significantly influence the choice for the surgical procedure nor the surgical outcome.

#### Menopausal age and comorbidity

The majority of patients having endometrial cancer were postmenopausal (87%). Cardiac comorbidity in the medical history was found in 42 patients (45%) and pulmonary comorbidity in 6 patients (6%). Sixteen patients were known with diabetes mellitus and 4 patients with a coagulation disorder. Previous abdominal surgery was performed in 37 patients (39%). Comorbidity and previous abdominal surgery did not significantly influence the choice for the surgical procedure nor the surgical outcome.

#### CA125

According to the Dutch guidelines, CA125 analysis has not to be performed routinely. In 86 (91%) of all patients the CA-125 serum level was determined preoperatively, in the remaining cases extra uterine disease was not suspected. In 17 patients (18%) the CA-125 serum level was elevated: 4 patients in group 1 (22%), 4 patients in group IIIa (17%) and 9 patients in group IIIb (19%). Elevation of CA125 did not significantly influence the choice for the surgical procedure or the surgical outcome.

#### Pre-operative histopathology

In 89 patients (95%) tumour histology preoperatively showed a malignant tumour. Preoperative histo-pathology lacked in 2 patients who were primarily operated because of vaginal prolapse (n = 1) or urinary incontinence (n = 1). Preoperative histologic examination showed endometrioid adenocarcinoma in 78 patients (83%) and a different histopathology in 11 patients (12%).

In group I, a pipelle biopsy was done in 16 patients (89%). All patients diagnosed by pipelle biopsy were diagnosed having endometrioid adeno-carcinoma (grade 1: 63%, grade 2: 38%).

In group II 4 patients (80%) were diagnosed with endometrioid adenocarcinoma. In 1 patient the preoperative biopsy did not show any malignancy. However, postoperatively a grade 3 endometrioid adenocarcinoma was diagnosed. Of all perioperative biopsied malignancies, 3 were grade 1 tumours (60%) and 1 was a grade 2 tumour (20%).

In group III, preoperative histologic examination showed an endometrioid adenocarcinoma in 58 patients (82%) and serous carcinomas in 11 patients (15%). Of all endometrioid adenocarcinomas in group III, 24 were grade 1 (41%), 26 grade 2 (45%) and 17 grade 3 (29%) tumours.

Because of our selection criteria for laparoscopic surgery the percentages of serous carcinomas were significantly higher in group III compared to group I (p < 0.001) and group II (p < 0.001). Poor and moderately differentiated non-endometrioid tumours were more commonly found in group IIIb compared to group I (p < 0.001), group II (p < 0.001) and group IIIa (p < 0.001).

#### Surgery

**Fig. 2:**
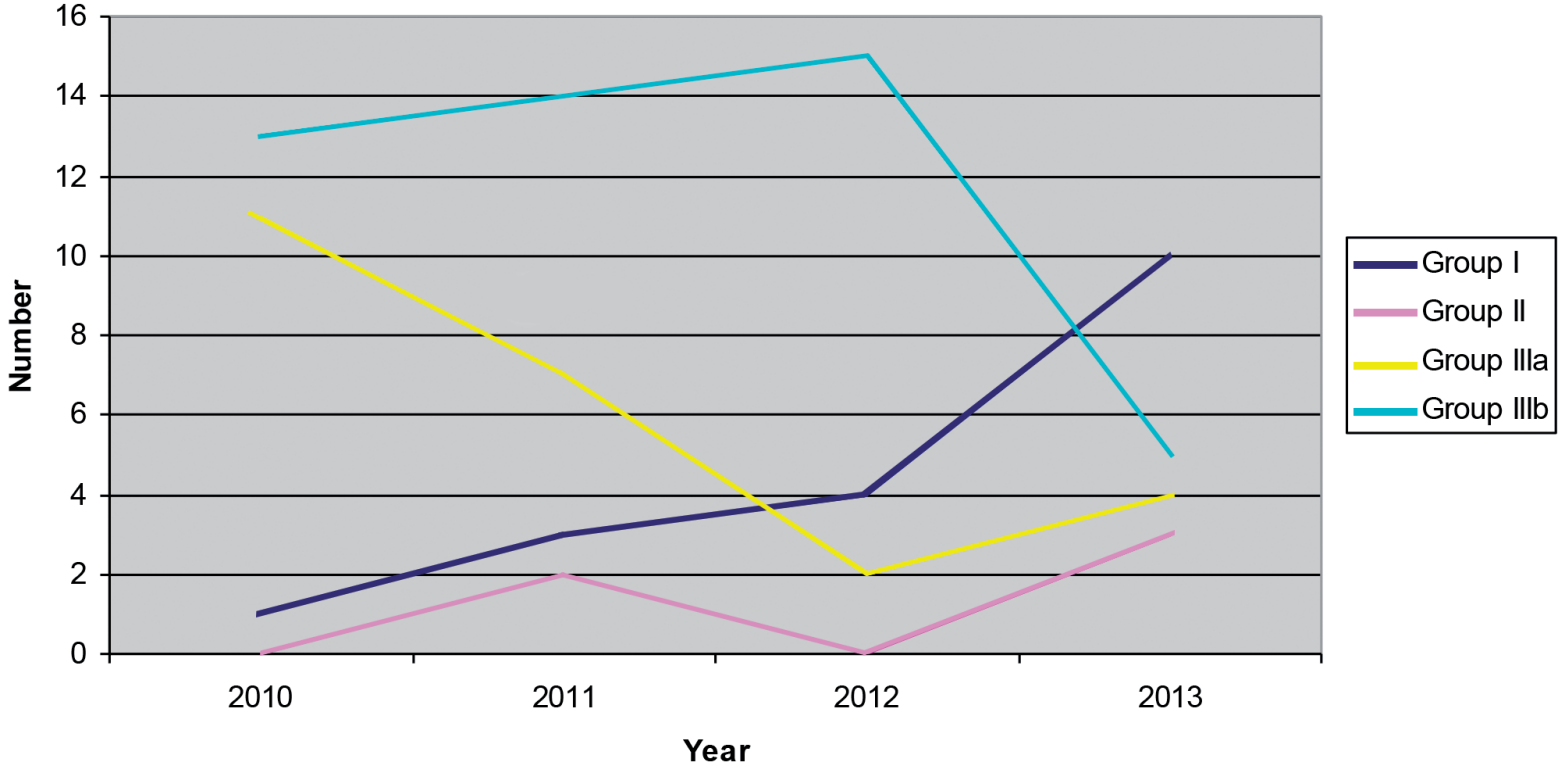
— 2013-2014: number of laparoscopies and laparotomies each year (Group I: Laparoscopy; Group II: Conversion after laparoscopy; Group IIIa:Laparotomy in low grade endometrial cancer; Group IIIb: Laparotomy in high grade endometrial cancer).

The gynaecologists slowly got used to the laparoscopic technique: the percentages of patients in group I increased and the percentages of patients in group III (a) decreased significantly over the years (p = 0.007) (Fig. 2).

**Fig. 3:**
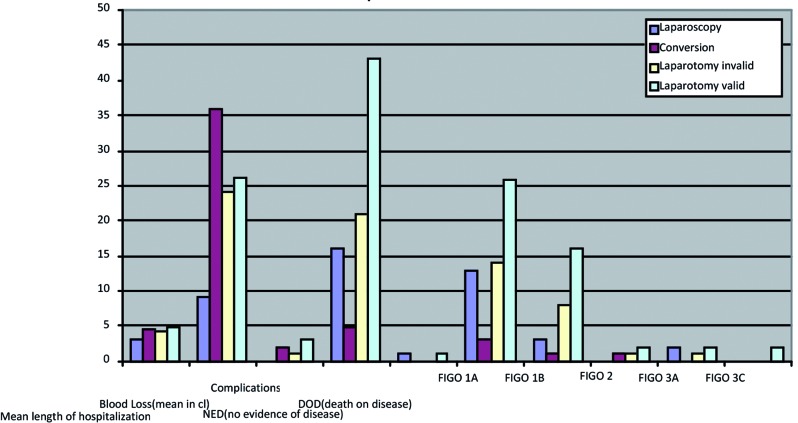
— Clinical relevant post-operative baseline characteristics. The left part of this figure shows different surgical procedures (laparoscopy is group I; conversion is group II, laparotomy invalid is group IIIa and laparotomy valid is group IIIB) in respect to postoperative baseline characteristics in patients that were operated because of endometrial cancer in this study (length of hospital stay, blood loss, overall complications, follow up). The right part shows these different surgical procedures in respect to FIGO stages of endometrial cancer.

The mean perioperative blood loss in all patients was 228.5 ml (95%-CI 19.1-386.4). The blood loss observed in the different groups was 91.1 ml (95%- CI 34.3-104.0), 359.8 ml (95%-CI 50.3-885.3), 242.5 ml (95%-CI 189.8-284.8) and 264.2 ml (95%- CI 236.9-298.0) for Group I, II, IIIa and IIIb respectively. The blood loss in group I is significant lower than in group IIIa (p = 0.002) and group IIIb (p < 0.001) (Table II, Fig. 3).

**Table II:** — Peri- and postoperative baseline characteristics in patients that were operated because of endometrial cancer in this study (laparoscopy = group I; conversion = group II; laparotomy invalid = group IIIa; laparotomy valid = group IIIB).

		Laparoscopy (n = 18)	Conversion (n = 5)	Laparotomy (n = 71)		p-value
				Laparotomy invalid (n = 24)	Laparotomy valid (n = 47)	
Postoperative type of tumour						0.55
	Endometrioid Adenocarcinoma	17 (94.4%)	5 (100%)	23 (95.8%)	34 (72.3%)	
	Serous Carcinoma	0	0	0	6 (12.8%)	
	Clear Cell Carcinoma	0	0	1 (4.2%)	6 (12.7%)	
	No malignancy	1 (5.6%)			1 (2.1%)	
Postoperative grade of tumour						0.01
	Good	10 (55.6%)	4 (80.0%)	12 (50.0%)	7 (12.8%)	
	Moderate	7 (38.9%)	1 (20.0%)	7 (29.2%)	23 (48.9%)	
	Poor	0	0	5 (20.8%)	16 (3.0%)	
	No malignancy	1 (5.6%)			1 (4.3%)	
Mean uterus weight in grams (95%-CI)		161.0 (99.9- 221.5)	338.9 (10.2- 402.7)	211.5 (113.0- 323.9)	196.9 (151.1- 260.7)	< 0.30
Uterus weight						0.92
	< 300 grams	13 (72.2%)	3 (60.0%)	20 (83.3%)	40 (85.1%)	
	> 300 grams	2 (11.1%)	1 (20.0%)	3 (12.5%)	6 (12.8%)	
	Unknown	3 (16.7%)	1 (20.0%)	1 (4.2%)	1 (2.1%)	
FIGO stage						< 0.03
	IA	13 (72.2%)	3 (60.0%)	14 (58.3%)	25 (53.2%)	
	IB	3 (16.7%)	1 (20.0%)	8 (33.3%)	16 (34.0%)	
	II	0	1 (20.0%)	1 (4.2%)	2 (4.3%)	
	IIIA	2 (11.1%)	0	1 (4.2%)	2 (4.3%)	
	IIIC	0	0	0	2 (4.3%)	
Blood loss, mean in ml (95%-CI)		91.1 (34.3- 104.0)	359.8 (385.3- 885.3)	242.5 (189.8- 284.8)	264.2 (236.9- 298.0)	< 0.01
Mean length of hospitalization, days (95%-CI)		3.1 (2.1-3.4)	4.6	4.4 (3.7-5.0)	4.9 (4.2-5.3)	< 0.01
Complications		0	2 (40.0%)	1 (4.1%)	3 (6.4%)	0.78
Relapse		0	0	2 (8.3%)	1 (2.1%)	0.48
Follow up						0.79
	NED	15 (83.3%)	5 (100%)	21 (87.5%)	43 (91.5%)	
	AWD	0	0	2 (8.3%)	1 (2.1%)	
	DOD	1 (5.6%)	0	1 (4.2%)	1 (2.1%)	
	DOC	2 (11.1%)	0	0	2 (4.3%)	

The mean length of postoperative hospitalization was 4.3 days (95%-CI 2.6-6.1). The mean length of hospitalization after surgery was lower in group I versus group II (p = 0.020), group IIIa (p = 0.003) and group IIIb (p < 0.001). (Table II, Fig. 3)

Postoperative complications occurred in 6 patients (6%). Of all primary laparotomies, 4 patients had complications (10.5%). Two patients (2%) had a postoperative infection cured with antibiotic treatment, 1 patient (1%) had a visual hallucination 2 days postoperatively which was cured with medication and another patient (1%) had a platzbauch postoperatively. There were no complications in the laparoscopic group. In the conversion group there where 2 complications (40%), one patient had an intra-operative sigmoid lesion with an uneventful recovery after suturing and one patient had wound healing problems which resolved with an expectant policy. (Table II, Fig. 3) There where significantly more complications in group II and III versus group I.

#### Uterine weight

Overall, the mean uterus weight was 202.8 grams (95%-CI 19.1-386.4). The mean uterus weight in Group I, II, IIIa and IIIb were respectively 160.7 grams (95%-CI 99.9-221.5), 196.3 grams (95%-CI -10.2-402.7), 218.5 grams (95%-CI 113.0- 323.9) and 205.9 grams (95%-CI 151.1-260.7). The preoperative estimation of uterine seize by gynaecologic examination or vaginal ultrasound did not significantly influence the choice for the surgical procedure. A large uterus or “too little space” was the reason for conversion in 5 patients.

#### Histopathology

Postoperatively, discrepancy in histopathology in the uterine specimen versus the uterine biopsy was seen in 29 patients (33%). After hysterectomy, 79 tumours were classified as endometrioid adenocarcinomas (84%), serous carcinomas (6%) and clear cell carcinomas (7%). Two patients did not have shown any residual carcinoma in the operative biopsy (one patient in group I and one in group IIIb).

In group I, one patient did not show any residual carcinoma in the operative biopsy. Well- differentiated carcinomas were seen in 10 patients (50%) and grade 2 endometrioid adenocarcinomas in 7 patients (39%).

In group I, the endometrioid carcinomas were grade 1 in 4 patients (80%) and grade 2 in 1 patient (20%).

In group III, twelve tumours were grade 1 (52%), 7 grade 2 (30%) and 5 grade 3 (22%).

In group III, endometrioidtumours were adenocarcinomas in 34 patients (72%), serous carcinomas in 6 patients (13%) and a clear cell carcinoma in 6 patients (13%). One patient did not show any residual carcinoma in the operative biopsy.

The percentages of poor and moderate differentiated and non-endometrioidtumours were significantly higher in group IIIb compared to group I (p < 0.001) and group II (p < 0.001).

#### FIGO Stage

All patients were classified according to the FIGO guidelines 2009 (IKNL, 2011). Overall, 56 patients (60%) were staged as FIGO stage IA, 28 patients (30%) as FIGO stage IB, 4 patients as FIGO stage II, 5 patients as FIGO stage IIIA and 2 patients as FIGO stage IIIC.

FIGO stage IA was signi cantly more seen in group I compared to group IIIa (p = 0.032) and groupIIIb (p = 0.004) (Table II, Fig. 3).

#### Follow up

Data were completed until 01-04-2015 with a median follow up of 41 months.

Overall, 7 patients died. Three of them died of endometrial cancer (one in group I; one in group IIIa and one in group IIIb). In group IIIb, two patients died because of cardiac and renal co-morbidity. In group I, one patient died due to a metastatic melanoma and in one patient the cause of death remained unclear.

During follow up, three patients (3%) relapsed. They all underwent a laparotomy. All of them were treated with adjuvant radiotherapy accordingly to the national guidelines and protocols. In group IIIa, two patients presented with vulvar metastases (n = 1) and one patient also had metastases in the top of the vagina (n = 1). In group IIIb, one patient had lung and abdominal metastases (Table II).

## Discussion

The incidence of endometrial cancer is slowly increasing (Bijen et al., 2010; Mourits et al., 2010; Barnett et al., 2011; Havrilesky et al., 2011; Obermair et al., 2012; Janda et al., 2012). This may be caused by changes in reproductive behaviour, an expanding number of obese women and the use of hormonal therapy. Indications for a laparoscopic procedure in gynaecologic cancer are based on tumour histology and tumour grade. Many studies have shown that laparoscopy is a safe procedure in the treatment of endometrial cancer (Mourits et al., 2010; Bijen et al., 2010; Barnett et al., 2011; Havrilesky et al., 2011; Obermair et al., 2012; Janda et al., 2012). It is also known that laparoscopic procedures are associated with less pain and a shorter postoperative hospital stay compared to laparotomies (Kanasaki et al., 2012; Oride et al., 2012).

In OMC laparoscopy in women with endometrial carcinoma is performed since 2010. We discussed the first experiences and implications of this new treatment strategy in gynaecologic oncologic surgery. Even though the first publications of treating endometrial cancer laparoscopically dates from 1993, it is shown that the introduction of a new technique is not immediately implemented and a lot of resistance may occur. Implementation requires confidence of medical specialists and their willingness to change routine processes.

Gynaecologists who are specialised in oncology are merely not the ones who are specialised in minimal and/or laparoscopic surgery. Because minimal invasive surgery is another subspecialisation in The Netherlands, these gynaecologists were more used to and familiar with these surgical techniques. The synergy between both subspecialists surely influenced the implementation of laparoscopy in the gynaecological oncologic practise. Therefore we believe that the fellowship gynaecologic oncology has to integrate laparoscopic surgery.

In 2009, Bijen et al. reported on the lack of consensus considering the safety of using laparoscopic surgery in patients with well and moderate differentiated tumours of endometrial carcinoma. Poor differentiated tumours are, because of surgical staging, operated by an open procedure (Bijen et al., 2009; Briet et al., 2009).

In 2012 two and in 2013 six moderately differentiated tumours were also operated by laparoscopy in the OMC. Moderately differentiated endometrioid adenocarcinomas became an indication for laparoscopic surgery.

At the end of the study period, laparoscopies were significantly more often performed than laparotomic surgeries. Full implementation of laparoscopic procedures in (low risk) endometrial cancer takes its time and brings new discussions. If we take a look at the 71 patients in group III, 19 patients could have been planned for a laparoscopic procedure retrospectively.

By multivariate logistic regression, besides the histopathology and grade of the tumour, no other independent factors for the choice between laparoscopy and laparotomy could be found (Table III).

**Table III:** — Multivariate logistic regression: odds of indications for planning the right surgery based on the protocol or not.

	OR	95%-CI	p-value
BMI (> 25 kg/m2)	1.150	0.257-5.144	0.855
Menopausal status	0.476	0.071-3.184	0.444
Cardiac history	1.668	0.488-5.697	0.414
Pulmonal history	4.477	0.476-42.102	0.190
Diabetes Mellitus	0.369	0.053-2.541	0.311
Coagulation disorder	3.520	0.329-37.647	0.298
Previous abdominal surgery	1.390	0.432-4.471	0.581
Serum level CA-125 (> 21 U/ml)	0.155	0.015-1.587	0.116

Papadia et al. (2009) concluded that frozen sections underestimate the need for surgical staging in endometrial cancer patients. We used to do staging by open laparotomy. Because staging for endometrial cancer is common when open surgery is performed we do not expect that indications for laparoscopy will rise in the near future.

Postoperatively less blood loss and a shorter hospital stay were seen in group I compared to the other groups. This is in line with the review of Carter et al. (2011) showing significantly more blood loss in patients undergoing laparotomy compared to laparoscopy and a reduced hospital stay in all patients undergoing a laparoscopic hysterectomy.

The clinical relevance of small differences in blood loss may be doubted. However in the view of costs and patient satisfaction the advantage of a shorter hospital stay is absolutely relevant.

The mean age of our patients was around 65 years of age. It was previously reported that laparoscopic surgery in patients over 65 years old with gynaecologic disease is a safe procedure (Kanasaki et al., 2012; Oride et al., 2012).

Obesity is a cofactor in the aetiology of endometrial cancer. Looking at the different patient characteristics such as weight, age and other co-morbidities, there was no significant difference between the different groups. Bhandari et al. (2014) also showed that laparoscopic hysterectomy in obese patients is a safe procedure with no significant difference in blood loss. Hauspy et al. (2010) reported that morbid obesity is a limiting factor for the feasibility of complete laparoscopic staging. Due to the many complications in obese patients after laparotomy, we recommend laparoscopy in all endometrial cancer cases unless there are strict indications for open surgery.
